# Strategies to successfully prevent COVID-19 outbreak in vulnerable uro-oncology patient population

**DOI:** 10.1007/s15010-022-01775-1

**Published:** 2022-02-24

**Authors:** Alexander Tamalunas, Melanie Schott, Troya Ivanova, Severin Rodler, Volker Heinemann, Christian G. Stief, Jozefina Casuscelli

**Affiliations:** 1 Department of Urology, University Hospital, LMU Munich, Marchioninistr. 15, 81377 Munich, Germany; 2 Department of Medicine III, University Hospital, LMU Munich, Munich, Germany; 3 Comprehensive Cancer Center, University Hospital, LMU Munich, Munich, Germany

**Keywords:** COVID, Uro-oncology, Urology, Oncology, Pandemic, Prevention

## Abstract

**Purpose:**

As COVID-19 pandemic persists with variants, and despite effective vaccination campaigns, breakthrough infections surge. We implemented strategies to protect vulnerable patients of the uro-oncologic outpatient clinic. We adopted proactive non-symptomatic risk reduction measures, which include non-symptomatic testing requirements for both patients and health care professionals (HCP), intensified patient tracing and contact reduction by implementation of digital health options. Here, we present our best practice example to safely guide oncology professionals and patients with metastasized genitourinary cancers through the current and future pandemics.

**Methods:**

Solely for this purpose, we created a registry of collected data (current telephone numbers, e-mail addresses, vaccination status). We collected a nasopharyngeal swab from every patient upon presentation for treatment. We implemented bi-weekly RNA-PCR assay tests for HCP with patient contact, and limited personal contact at our facility through digital patient consultations.

**Results:**

We started implementing our COVID prevention model at the beginning of the second wave in September 2020 and included 128 patients with urologic malignancies requiring systemic treatment. After COVID vaccination became available in December 2020, all of our HCP were fully vaccinated within 6 weeks and 97% of our patients (125/128) within 9 months. We performed 1410 nasopharyngeal swabs during in-house visits, thereby detecting two COVID-19 infections among our patients, who both survived and successfully continued treatment. To further reduce personal contact, half of our consultations were fully operated digitally, with 76% (97/128) of our patients participating in our digital health offers.

**Conclusion:**

The willingness of patients and HCPs to participate in the study allowed us to implement strict standards to prepare for the ongoing and future pandemics in outpatient cancer units. Next to general preventive measures such as frequent hand disinfection, wearing facial masks, and keeping distance, an important measure to protect vulnerable uro-oncology patients is the capability to perform virus genome sequencing to trace transmission chains.

## Introduction

Severe acute respiratory syndrome coronavirus 2 (SARS-CoV 2) was first identified in humans in December 2019. Spreading rapidly, it led to the ongoing coronavirus disease 2019 (COVID-19) pandemic, critically challenging the world’s finite health care system [1, 2]. Oncology professionals have been facing a difficult task ever since: keeping a vulnerable patient population safe, who is—in general—at high risk of severe events. In this context, it has been speculated that patients with metastatic cancers might be prone to higher risk of infections with SARS-CoV 2, due to frequent outpatient visits [3]. While increased susceptibility to SARS-CoV 2 infection in cancer patients was controversially discussed at the beginning of the pandemic, data now show that there may be differential susceptibility relative to tumor type [3–6]. However, when cancer patients suffer from COVID-19, the mortality rate of hospitalized patients is much higher than compared to non-oncology patients, especially in patients with hematological tumors or metastasized disease [7].

As a tertiary health care facility and academic cancer center, we have been on the forefront of Germany’s response to COVID-19 from the very beginning. The uro-oncologic outpatient care facility at our institution was affected in March 2020 by the outbreak of COVID-19, which forced us to implement changes in our treatment and follow-up procedures [8]. COVID-19 pandemic persists with variants despite effective vaccination campaigns. Breakthrough infections surge in the autumn of 2021, while in Germany free testing was abolished in October 2021 amid a rapidly rising case rate to incentivize people to get vaccinated [9, 10]. With most of our patients metastasized and have to keep up with treatment intervals, we implemented strategies to protect vulnerable patients of the uro-oncologic outpatient clinic. Having learned from our early encounter with an in-house COVID-19 outbreak, we adopted proactive non-symptomatic risk reduction measures [8]. Our standard operating procedures included non-symptomatic testing requirements for both patients and health care workers, intensified patient tracing and contact reduction by implementation of digital health options.

Here, we present our best practice example on how to safely guide oncology professionals and patients with metastasized genitourinary cancers through the current and future pandemics.

## Methods

### Patient data registry

Patients undergoing systemic therapy for genitourinary cancers at the uro-oncologic outpatient clinic (Ludwig-Maximilians-University, Munich, Germany) were prospectively included. Starting in September 2020 in preparation for the second COVID-19 wave and solely for this purpose, we created a database as registry of collected data. To ensure maximum efficiency in communication, we asked all patients to provide an emergency contact telephone number and e-mail address. We gathered information on patients’ vaccination status (i.e., influenza, pneumococci), including the vaccination status of all household members. From January 2021 on, vaccination status included COVID-19 vaccinations, and starting in September 2021, we promoted COVID-19 booster vaccinations. Patient data registry also included date of visit, treatment line, treatment medication, result of nasopharyngeal swab for COVID-19 screening and use of COVID-19 tracking apps.

### Restructured patient in-house visits

We instructed patients to contact us in advance to their in-house visit, if they felt any COVID-19 or flu-like symptoms. Depending on symptoms mentioned, patients were further instructed to get a SARS-CoV 2 RNA-PCR assay prior to their scheduled visit, or visits were re-scheduled. In addition, patients were handed a symptom questionnaire at the beginning of each visit to screen for COVID-19 symptoms. The questionnaire contained eight questions, which included (1) fever > 38 °C, (2) shortness of breath/frequent coughing, (3) nasal congestion, (4) sore throat, (5) fatigue, (6) sense of taste and smell impairment (7) SARS-CoV 2 RNA-PCR assay test within the past 14 days, and (8) exposure to a person with COVID-19. Admittance was only granted, if patients selected none of the above, or reported a negative SARS-CoV 2 RNA-PCR assay test. To minimize exposure of household members, a companion was only allowed if absolutely necessary, i.e., for patients requiring assistance or a translator.

### Periodic and deliberate COVID-19 screening and awareness

In addition to the restructured in-house visits, we constantly raised awareness for COVID-19 within our uro-oncology patient cohort. We collected a nasopharyngeal swab from every patient upon presentation for treatment. Testing for SARS-CoV 2 was performed at the department of Laboratory Medicine, LMU Munich, Munich, Germany by polymerase chain reaction (PCR) for SARS-CoV-2-RNA N-Gene 1, and also included detectable variants. Furthermore, HCP with direct patient contact were invited to undergo bi-weekly RNA-PCR assay tests.

### Algorithm for SARS-CoV 2 positive persons

**Fig. 1:**
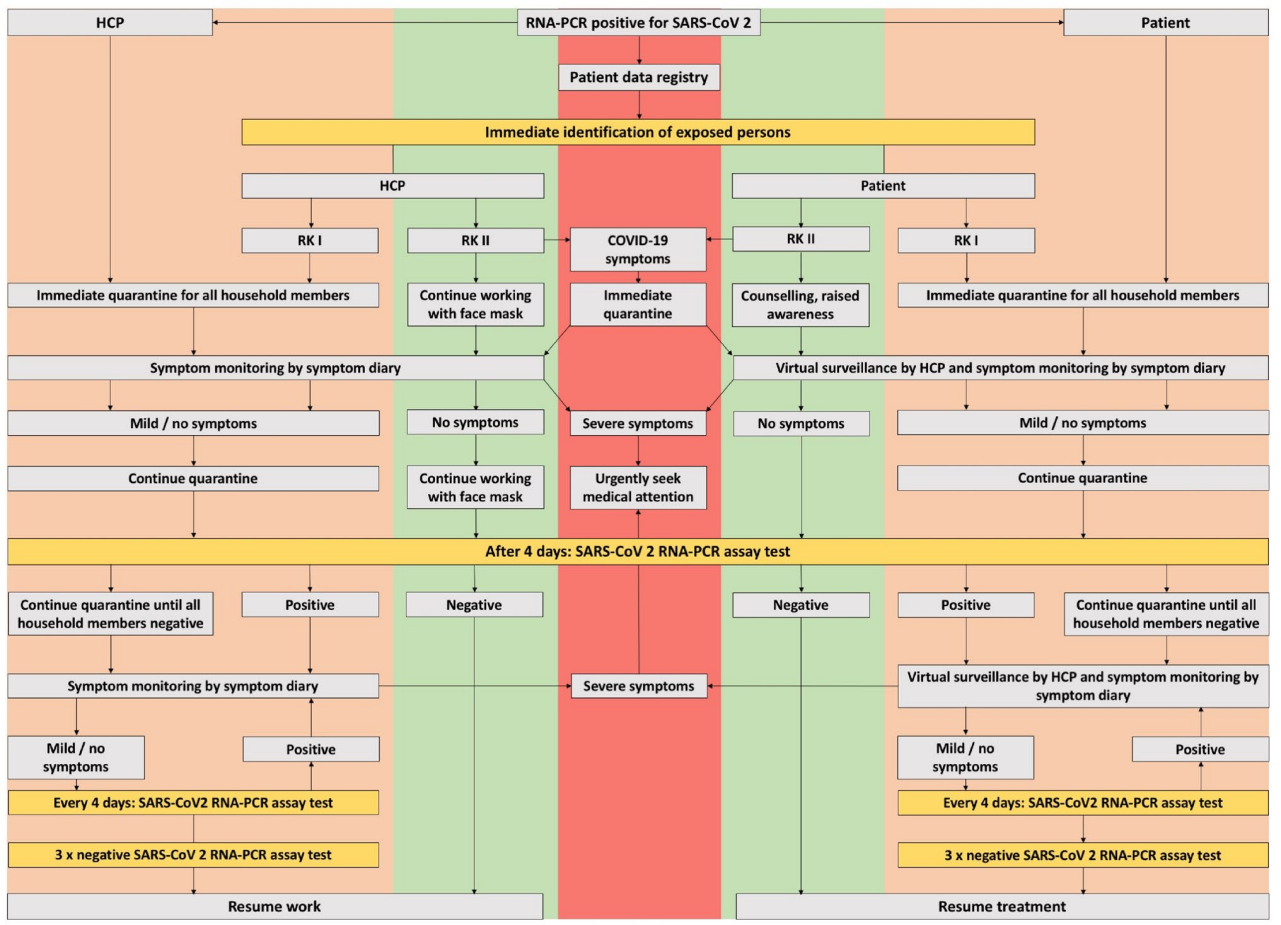
Algorithm by which all staff and patients from the uro-oncology academic center proceeded after either a patient or member of staff tested positive (+) for SARS-CoV 2 by RNA-PCR assay. Not only did we follow-up and include patients and personnel who tested positive for SARS-CoV 2, but also included tracking the infection chain by exposure using data gathered in our patient data registry. Only after symptoms sufficiently ceased and SARS-CoV 2 positive persons tested negative three times within 14 days, treatment or working at the uro-oncologic academic center could be resumed. *SARS-CoV 2* severe acute respiratory syndrome coronavirus 2, *PCR* polymerase chain reaction, *RK I/II* Robert-Koch-Institute exposure risk grade I/II, *COVID-19* coronavirus disease 2019

When implementing regular and deliberate testing of patients and HCP for SARS-CoV 2, developing an algorithm became mandatory. In case a member of staff or patients tested positive on their nasopharyngeal swab, we followed the protocol depicted in Fig. 1. The protocol was developed by *Robert Koch Institute* (RKI) [11].

### Implementing digital health

Following the COVID outbreak in our outpatient clinic in March 2020, we offered telehealth solutions for patient consultations, which allowed patients to reduce personal contact at our facility, whenever feasible [12].

## Results

**Fig. 2:**
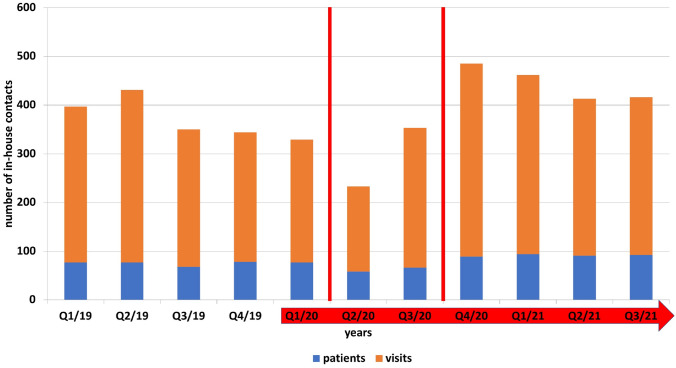
Number of in-house visits and total patients by quarter-yearly analysis. The red arrow marks the beginning of COVID-19 pandemic. The vertical lines mark first and second lock-down of public life on March 17 2020 and November 2 2020, respectively

From implementing our COVID data registry system in September 2020–September 2021, a total of 128 patients with malignancies of the urothelium (*n* = 41), kidney (*n* = 35), or prostate (*n* = 52) underwent cancer-specific therapy in our specialized uro-oncology outpatient clinic. Patients received chemotherapy (*n* = 37), immunotherapy (*n* = 55), tyrosine kinase inhibitors (*n* = 6), secondary androgen-deprivation following chemotherapy (*n* = 26), and four patients received Bacillus Calmette-Guerin (BCG). The remaining patient characteristics are displayed in Table 1. During this time, 13 patients (10.2%) died of causes associated to their respective malignancies. Patients receiving intravenous systemic therapy and their respective visits are displayed by quarter-yearly evaluation in Fig. 2. Pre COVID-19, we report a median of 77 patients with a median of 301 visits per quarter, during the first wave of COVID-19 and following the COVID-19 outbreak in our specialized outpatient uro-oncology clinic we outsourced patient and reduced the number of patients being treated in-house to a quarterly median of 66 with a median of 252 visits per quarter, thereby reducing patients and in-house visits by 14.3 and 16.3%, respectively. However, and to keep up our high-quality standard of care, we successfully upheld the median visit per patient at 3.81 quarter-yearly visits compared to 3.90 pre-COVID-19. After implementing our COVID data registry system and with COVID vaccinations available from December 2020 on, we became confident in resuming our in-house treatments and even taking on new patients. We increased the number of patients with intravenous antitumoral therapy by 38.7 and 18.8% compared to COVID first wave and pre-COVID, respectively, thereby also increasing median visits per quarter from 252 during COVID and 301 pre-COVID to 346. Within 6 weeks of COVID vaccination program start in Germany all of our uro-oncology professionals were fully vaccinated, and within 9 months 97.3% (125/128) of our patients were fully vaccinated. We report, that 36.7% (47/128) of all patients have received influenza vaccination, 39.4% (41/128) and 14.8% (19/104) of the ≥ 60 year-old patient cohort (41/104) have received either pneumococcal vaccination or both, and only 35.9% (46/128) have not received any of the two vaccinations. Interestingly, only 18.8% (24/128) used the German *Corona Warn App* for tracking infection chains [13]. From September 2020 to September 2021, we performed 1410 nasopharyngeal swabs for RNA-PCR assay tests for patients, with only two patients testing positive for COVID-19 (0.14%, 2/1410) in late December 2020. Both patients had no symptoms prior to the nasopharyngeal swabs, and no contact to each other. HCP in direct contact with patients performed bi-weekly nasopharyngeal swabs, with only one uro-oncology professional testing positive for COVID-19 in January 2021 only hours before the scheduled vaccination appointment. Thus, all SARS-CoV 2 positive RNA-PCR assay test results occurred during the peak of the second COVID-19 wave in the winter of 2020/2021, before vaccinations became widely available. With the introduction of telehealth offers, we specifically scheduled 50% of our patient consultations as virtual meetings, thereby reaching 75.8% (97/128) of our patients on a regular basis.

**Table 1:** Patient characteristics and vaccination status

Age		
Median	70.2	
IQR	62–78	
Total (*n* = 128)	*n*	%
Cancer		
Urothelium	41	32.1
Kidney	35	27.3
Prostate	52	40.6
Therapy		
Chemotherapy	37	28.9
Immunotherapy	55	42.9
Tyrosine kinase inhibitors	6	4.7
Sec. androgen-deprivation	26	20.3
BCG	4	3.2
Sex		
Male	89	69.5
Female	39	30.5
Comorbidities		
Hypertension	59	46.1
Obesity	34	26.6
Diabetes	16	12.5
Renal disease	24	18.8
Vaccination status		
Influenza	47	36.7
Pneumococci^a^	41	39.4
Influenza + pneumococci^a^	19	14.8
COVID-19	125	97.3
None	46	35.9
Corona warn app		
Yes	24	18.8
No	104	81.2
Digital health participation		
Yes	97	75.8
No	31	24.2
COVID-19 infection		
Yes	2	1.6
No	126	97.4

*BCG* Bacillus Calmette-Guerin, *COVID-19* coronavirus disease 2019

^a^Percentage given for the population ≥ 60 years (*n* = 104)

## Discussion

For the past 18 months, health care professionals around the world have been facing one of the biggest health care challenges of all time: COVID-19. Apart from the patients actually suffering from COVID-19, navigating all other patients through the pandemic, became a mandatory task for oncology professionals. This exceptionally vulnerable patient population is fundamentally dependent on a functioning health care system, with health care providers working closely together on a multidisciplinary level to ensure maximum patient safety while keeping up necessary treatments. Being hit hard and early during the first wave of COVID-19 in March 2020, we were forced to prepare for a possible second wave during the fall and winter of 2020/21 [8]. While, in the beginning, our preparations were rudimentary and focused on outsourcing patients for a limited time to ensure their wellbeing, we now report data from the past year and lessons learned through a fundamentally restructured uro-oncology ward to continue providing high-standard health care providing a safe environment for patients and staff alike.

Our patient data registry formed the most central part in our effort to regain the ability to efficiently function as an uro-oncologic academic center. It allowed us to centralize critical information about our patients as to when and where they frequented our uro-oncology outpatient clinic. The utmost effort was put into assuring that whenever patients or HCP tested positive for SARS-CoV 2, identification of in-house contact persons could be achieved within minutes, thereby successfully preventing further possible spread of the virus. Whenever a member of staff or patient tested positive for SARS-CoV 2, we followed the algorithm shown in Fig. 1. The algorithm is based on recommendations by the German center for disease control, *Robert Koch Institute* (RKI), and was modified to make use of profit from our patient data registry [11]. The most critical part to sustain an efficiently working outpatient clinic was by identifying contact persons and by defining a grade of exposure. So instead of retrospectively adding all contact information and possible contact persons, thereby losing time and binding resources, we already had all the current and crucial information contained in our updated patient data registry. Exposed persons were defined as persons with “direct/close” (risk category 1, RK1) contact and “indirect” (risk category 2, RK2). While RK1 contact was defined as face-to-face for > 15 min without surgical face masks, RK2 contact was defined as any other form of contact > 15 min, while wearing a surgical face mask [11]. HCP with RK2 exposure were allowed to continue working while always wearing a medical face mask, while patients with RK2 exposure were intensely counseled by oncology professionals and scheduled for a SARS-CoV 2 RNA-PCR assay test after four days. All persons with RK1 exposure and SARS-CoV 2 positive persons were immediately ordered to quarantine. Naturally, all positive and exposed persons were urged to seek immediate medical care when experiencing severe symptoms. Respectively, HCP and patients were only allowed to resume working at the hospital or continue treatment after providing three negative SARS-CoV2 RNA-PCR assay test results within 14 days.

However, we also gathered patients’ vaccination status to analyze the impact of co-vaccination on the susceptibility of SARS-CoV 2. In Germany, the Standing Committee on Vaccination (*Ständige Impfkommission;* STIKO) recommends vaccination regimes and adjusts recommendations, if scientific data commands it. Currently, only 24.2% of the population ≥ 60 years of age have been vaccinated against pneumococci and only 38.8% of the total population against influenza [14]. While our data on influenza vaccination status corresponds to this, we found a higher percentage of the ≥ 60 year-old patient cohort to have either received pneumococcal vaccination, influenza vaccination, or both. The discrepancy clearly marks our efforts informing and encouraging patients to get vaccinated and, of course, the vulnerability and heightened attention of oncologic patients for infectious diseases. A synergistic effect of pneumococcal and influenza vaccination and their respective infectious diseases on contracting SARS-CoV 2 and developing serious COVID-19 is hypothetically obvious and has been discussed [15]. However, and due to the fortunately low infection rate with COVID-19 in our uro-oncologic patient cohort, we can neither confirm nor dismiss an effect of co-vaccination on susceptibility of contracting SARS-CoV 2 or developing COVID-19.

Our strategy to protect patients from COVID-19 further included limiting admissions to patients only, and allowing one companion for incapacitated patients. Patients and companions had to fill out a 87 item questionnaire at the beginning of their visit and were only admitted after answering as described in the methods section. Additionally, oncology patients were allocated strict time slots and prioritized upon arrival to ensure maximum efficiency and minimum contact to other patients or HCP other than treating physicians and nurses. However, and to track possible infection chains, we also registered each visit in our patient data registry, allowing us to immediately link all patients and personnel to each other, who were treated at our facility on the same day. Thus, limiting personal contact and potential spread of SARS-CoV 2 at our uro-oncology outpatient clinic. By following the established protocol, we were able to successfully prevent an outbreak during the three positive cases in December 2020 and January 2021.

From March 11th 2020, all persons in the hospital were required to wear surgical face masks, progressing to filtering facepiece 2 (FFP2) standard masks from January 18th 2021 on by ministerial decree [16]. All HCP constantly raised awareness of the strict face mask mandate and lead by example, granting no exceptions. As of October 6th 2021, FFP2 masks have become optional again, but surgical face masks remain mandatory in closed-space settings.

An early COVID-19 outbreak in the uro-oncology ward already prompted us to restructure our consultation regime as early as March 2020 [8]. We strictly limited personal contact to treating uro-oncology professionals only and aimed to decentralize patient care from our multidisciplinary academic center to primary and secondary care providers, while keeping treatment oversight. Patients were given the treating physician’s office e-mail address to allow virtual treatment monitoring and exchange of imperative data. Treatment monitoring was kept up by regular imaging intervals at a collaborating practice and special time slots for uro-oncologic patients at Department of Radiology, LMU Munich, Munich, Germany. Diagnostic blood workup was encouraged to be performed at a primary care provider no less than 48 h before presentation for antitumoral treatment, further limiting waiting periods to a minimum. Patient follow-up, symptom monitoring and treatment of side effects from antitumoral therapy were monitored virtually by frequent telephone calls and increased communication via e-mail. All documentation and patient data are transferred by current data protection laws via a secure server network. As reimbursement for telehealth offers has been implemented, we continue our effort to supply virtual patient consultations and only revert to personal consultations, if intensified counseling is mandatory. Overall, offering telehealth was a great success and patients adopted virtual counseling quickly regardless of age, and digital efforts will be continued at our facility in the future.

To ensure high-standard health care and a COVID-19 safe environment for patients and staff alike, and amid rapidly rising numbers of COVID-19 cases also among the vaccinated population, we will pursue our strict and effective protocol to overcome the impending third winter with COVD-19. Booster vaccinations will be promoted, and while HCP were administered their shot by September 2021, all our patients were encouraged to refresh their immune status by COVID-19 and current flu vaccinations. While general preventive measures, such as frequent hand disinfection, wearing facial masks, and keeping distance are still mandatory, the high rate of vaccinated patients in combination with regular SARS-CoV 2 RNA-PCR assay testing have surely contributed to the successfully low infection rate in our study cohort. Thus, and despite the controversial rule to abolish public free COVID-19 testing, we were able to ensure regular testing to our HCP and patients to avoid outbreak of breakthrough infections at our facility, as we deem this measure one of the most effective in battling the current pandemic.

Limitations include a single-center approach as tertiary health care facility and academic cancer center, and limited number of patients (*n* = 128). Naturally, results need to be verified in larger and multicenter patient cohorts in order to be able to include a recommendation and validate the efficacy of our proposed measures. However, we feel comfortable in presenting our successful best practice example on how to safely guide oncology professionals and patients with metastasized genitourinary cancers through the current and future pandemics.

## Conclusion

In conclusion, the willingness of patients and HCPs to implement and adopt strict standards allowed us to prepare for the ongoing and future pandemics in our outpatient cancer unit. Next to general preventive measures such as frequent hand disinfection, wearing facial masks, and keeping distance, an important measure to protect a vulnerable patient cohort is the financial and technical capability to perform virus genome sequencing to trace transmission chains.

## Data Availability

The data that support the findings of this study are available from the corresponding author upon reasonable request.
